# Thallium stimulates ethanol production in immortalized hippocampal neurons

**DOI:** 10.1371/journal.pone.0188351

**Published:** 2017-11-21

**Authors:** Laura Colombaioni, Massimo Onor, Edoardo Benedetti, Emilia Bramanti

**Affiliations:** 1 CNR Neuroscience Institute, Area della Ricerca CNR, Pisa, Italy; 2 National Research Council of Italy, C.N.R., Institute of Chemsitry of Organo Metallic Compounds-ICCOM, Pisa, Italy; 3 Hematology Unit, Department of Oncology, University of Pisa, Pisa, Italy; Oregon Health and Science University, UNITED STATES

## Abstract

Lactate and ethanol (EtOH) were determined in cell culture medium (CCM) of immortalized hippocampal neurons (*HN9*.*10e cell line*) before and after incubation with Thallium (Tl). This cell line is a reliable, *in vitro* model of one of the most vulnerable regions of central nervous system. Cells were incubated for 48 h with three different single Tl doses: 1, 10, 100 μg/L (corresponding to 4.9, 49 and 490 nM, respectively). After 48 h, neurons were “reperfused” with fresh CCM every 24/48 h until 7 days after the treatment and the removed CCM was collected and analysed. Confocal microscopy was employed to observe morphological changes. EtOH was determined by head space—solid phase microextraction -gas chromatography -mass spectrometry (HS-SPME-GCMS), lactate by RP-HPLC with UV detection. Tl exposure had significant effects on neuronal growth rate and morphology. The damage degree was dose-dependent. In not exposed cells, EtOH concentration was 0.18 ± 0.013 mM, which represents about 5% of lactate concentration (3.4 ± 0.10 mM). After Tl exposure lactate and EtOH increased. In CCM of 100 and 10 μg/L Tl-treated cells, lactate increased 24 h after reperfusion up to 2 and 3.3 times the control value, respectively. In CCM of 10 and 100 μg/L Tl-treated cells 24 h after reperfusion, EtOH increased up to 0.3 and 0.58 mmol/L. respectively. These results are consistent with significant alterations in energy metabolism, despite the low doses of Tl employed and the relatively short incubation time.

## Introduction

In the last years there is a growing concern about thallium (Tl) as a hidden geochemical hazard in different areas of the world with ‘natural’ contamination, which indicates potential toxicity for humans through geological pathways [1, 2]. Currently, chronic exposure to Tl seems to be a global phenomenon, as several cases of water contamination have been registered [3–7]. Recently, the European COST Action TD1407 included Tl in the list of technology-critical elements, with associated environmental impact and potential human health threats [8]. The maximum contaminant level of Tl in drinking water was fixed by U.S. EPA at 2 μg/L, with the goal of lowering it at 0.5 μg/L [9] and by China at 0.1 μg/L [10]. Thallium concentration in drinkable water is not regulated in most countries although the occurrence of Tl in drinkable water is not exceptional or rare [11]. Thus, chronic exposure may occur for long time at Tl concentration levels ranging from 0.1 to 100 μg/L [7, 12].

Thallium is a soft metal and the oxidation states are I and III. Thallium is more toxic to humans than mercury, cadmium, lead, copper, or zinc [2, 9].

Oral intake of 20–60 mg Tl/kg body weight is lethal within one week. The major symptoms of acute intoxication are anorexia, vomiting, mental depression, hair loss, cardiac and respiratory failure. The same symptoms are reported for chronic intoxication. The reference range for acute and chronic poisoning both for blood and urine is <1mg/L [13]. Thallium is absorbed through the skin and mucous membranes, it is distributed in all tissues and organs, including placenta and fetus in pregnant women. Tl accumulates in bones, kidney, central nervous system, hair and nails and it is excreted in the urine and feces. In urine Tl biological half-life is 3–8 days [2, 14] or 12.5 days [15]. In the case of chronic contamination the slow release from body tissues can determine its presence in urine samples above the reference values up to one year (time tested) after the interruption of the exposure [7, 16].

Tl is also known to bind to -SH groups of cytosol and membrane proteins (especially mitochondrial membrane proteins), interfering with many enzyme reactions (pyruvate kinase, ATPase, aldehyde dehydrogenase, proteins of cellular respiration) [17–20]. Since Tl^+^ and K^+^ are both univalent ions with similar ionic radii, Tl is able to interfere with potassium-dependent processes in the (Na^+^ /K^+^)-ATPase, in the stabilization of ribosomes and in muscle contraction [21, 22]. Thallium also affects the enzyme production and amino acid synthesis, the transport mechanisms and the mitotic process [21].

Although the neuronal Tl toxicity is well known [23, 24], at the present, no experimental data are available on Tl-induced alterations of human hippocampal neurons.

In this work we present the results of the determination of ethanol (EtOH) and lactate in cell culture medium (CCM) of immortalized hippocampal neurons (*HN9*.*10e cell line*). This neuronal cell line shares a large number of structural and functional features with primary hippocampal neurons and, consequently, it is a reliable *in vitro* model of one of the most vulnerable regions of central nervous system [25]. HN9.10e was chosen since it is a well characterized cell line [25], which allows us to reliably evaluate minute metabolic and functional alterations. It can be easily expanded and transfected with new engineered protein probes to monitor specific cellular functions in further studies [26–29]. Furthermore, in hippocampus the neurogenesis continues in adulthood [30] and as well as in the neocortex and striatum of many vertebrates, including humans [31–33]. An enhancement of the neurogenesis has been associated with learning and memory [34, 35], whereas it may diminish in case of pathologies, mood disorders, or stress [36, 37]. Thus, this is a significant and crucial phenomenon in CNS physiology, and therefore it is important to study the effects of toxic agents in proliferating neurons.

This region is particularly susceptible to the toxic action of drugs or poisons and it is also severely damaged by neurodegenerative diseases [38]. The choice of examining CCM instead of cell lysates or other matrices is due to the relative simplicity of measuring the variations of chemical composition in CCM.

EtOH was determined by head space—solid phase microextraction-gas chromatography -mass spectrometry (HS-SPME-GCMS), lactate by reversed phase liquid chromatography (RP-HPLC) with UV detector. EtOH is one of the most important volatile organic compounds (VOCs) and in our experiments it was the main, unexpected component of VOCs detected in the HS of CCM. In a recent review Filipiak et al. summarized the importance and the determination of VOCs in various body matrices to investigate physiological and pathophysiological processes and in human cell line cultures in order to investigate the metabolic pathways [39]. Only one paper has reported on the detection of EtOH in A549 lung cell lines [40]. Lactate was determined because it is a precursor of EtOH in cell metabolism.

Released ethanol and lactate were determined in CCM after 48 h incubation with three different single Tl doses: 1, 10, 100 μg/L (4.9, 49 and 490 nmol/L, respectively). These concentrations were much lower than those previously used for the treatment of other cell culture systems [41–44] and related to the concentrations recently found in drinking water of some contaminated regions [7, 12]. After 48 h exposure to Tl single dose, neurons were “reperfused” with fresh CCM every 24/48 h until 7 days after the treatment and the removed CCM was collected and analysed.

## Materials and methods

### Chemicals and reagents

EtOH (1.00980 EMD Millipore, absolute, for HPLC, ≥99.8%), L-Lactic acid (L-6402, 98%), Thallium chloride (224898) were purchased from Sigma-Aldrich-Fluka (Milan, Italy). Thallium compounds are toxic and were handled with caution in a ventilated fume hood, using appropriate protective clothing. Phosphoric acid for HPLC analysis was prepared from monobasic monohydrate sodium phosphate (BDH Laboratory Supplies, Poole, England) and phosphoric acid (V800287 VETEC ≥85%). Methanol for RP-HPLC was purchased from Carlo Erba (Rodano, MI, Italy). Preparation/dilution of samples and solutions was performed gravimetrically using ultrapure water (MilliQ; 18.2 MΩ cm^-1^ at 25°C, Millipore, Bedford, MA, USA).

Solid Phase Micro Extraction Fiber 85 um Carboxen/PDMS were employed for the preconcentration of volatile compounds in the HS.

### Cell culture and confocal imaging

The HN9.10e cell line was originally developed by Lee and Wainer by immortalization of murine hippocampal neuroblasts through the somatic cell fusion with N18TG2 neuroblastoma cells [25].

HN9.10e cells were grown in DMEM-F12 (1:1) medium HEPES buffered, supplemented with 2 mM L-glutamine, 50 UI/mL penicillin and 50 mg/mL streptomycin, at 37°C in humidified atmosphere containing 5% CO_2._ Cells, seeded at 20,000 cell/cm^2^ in culture flasks containing 5 mL of medium, were left in culture for 4 days before treatments in order to allow substrate adhesion and growth to an optimal 40–60% confluence. After this, they were incubated for 48 h in TlCl 1, 10 or 100 μg/L. In order to analyze morphology and growth rate, cells were vitally stained with 0.5 μM calcein-AM **(**Thermo Fisher Scientific), a cell-permeant dye typically used as aspecific cytoplasmic stain. Calcein-AM (non-fluorescent) is converted in live cells, by the intracellular esterases, into the green-fluorescent and membrane-impermeant calcein dye, that is retained inside cells. Calcein was imaged by confocal microscopy using as λ_ex_ the laser line at 488 nm of a TCS-NT Leica laser scanning confocal microscope (Leica Microsystems, Mannheim, Germany) equipped with 40x or 63x Plan Apo oil objectives.

The degree of cell confluence was evaluated by the automated measure of the ratio [surface occupied by cells / cell-free surface] with a dedicated routine of MATLAB scientific software (The MathWorks, Massachusetts, U.S.A.). The measurements have been performed in N = 3 independent experiments for each dose, n = 5 sampling fields (400 x 400 μm) in each experiment (15 total fields from N = 3 independent experiments). In initial conditions (t = 0), when the confluence value was 0.4, each field contained 150 cells (average value). Apoptotic and necrotic cells were distinguished on the basis of standard, well recognized morphological features [45, 46]. Apoptosis is characterized by cytoplasmic shrinkage, plasma membrane blebbing, without integrity loss, nuclear collapse and formation of pyknotic bodies of condensed chromatin. Necrotic cells, on the other hand, exhibit loss of membrane integrity, with cellular and nuclear swelling. Apoptotic and necrotic neurons have been counted in 15 total fields from N = 3 independent experiments.

For the analysis of EtOH and lactate by HS-GC-MS and HPLC, respectively, 5 mL of CCM were removed from the flasks and collected in 10 mL headspace vials (Agilent Technologies, Part No. 8010–0038). The vials were sealed with holed screw-caps equipped with teflon/silicone septa for use with the CombiPAL (Agilent Technologies, Part No. 8010–0139) and kept at -20°C until the SPME-HS-GC-MS and HPLC analysis. The transfer of such volumes was accomplished using adjustable pipettes and, for better precision, all aliquots were weighted. After 48 h exposure to TlCl, CCM was removed every 24/48 h and replaced with fresh medium. The removed CCM was collected and analysed. This sampling was regularly carried out up to 7 days after the initial Tl treatment.

### Analytical instrumentation and procedures

#### EtOH analysis by SPME-HS-GC-MS

EtOH was analyzed by SPME sampling of headspace (HS) in GCMS. An Agilent 6850 gas chromatograph, equipped with a split/splitless injector, was used in combination with an Agilent 5975c mass spectrometer. A CTC CombiPAL autosampler was employed for the SPME HS sampling. The vials were incubated at 50°C for 10 min. The SPME fiber was exposed for 5 minutes in the HS and injected in splitless mode into the gas chromatograph. The fiber was flushed inside the injector with helium at 300 mL/min for 15 minutes for cleaning (no carry-over problems were encountered). The inlet liner (1 mm internal diameter) was held at 280°C and the injection was performed in splitless mode (15 s splitless time) in the column (helium flow rate 1 mL/min). Compounds were then separated on a high polarity column (DB-FFAP; 60 m length;: 60 m; terephtalic acid modified carbowax stationary phase; 0.25 mm inner diameter; 0.5 μm coating) using the following temperature program: 10 min held at 30°C, 4°C/min up to 240°C held for 15 min (55 min total runtime). The temperature of the transfer line was set at 240°C. After GC separation, EtOH was ionized in positive EI. The MS acquisition was performed in total ion chromatography (TIC) and in selected ion monitoring (SIM) modes. TIC allowed us the identification of the EtOH, whereas SIM was implemented for quantitative purposes by monitoring m/z of 45, 46 and 31 (100 ms dwell time).

For the calibration of the instrumental response, 5 mL of 0, 0.023, 0.046, 0.23, 0.46 and 2.3 mM EtOH (primary standard solution) were introduced in 10 mL headspace vials (Agilent Technologies, Part No. 8010–0038). For EtOH determination the dynamic linear range (DLR) was 0.002–2.5 mM, 0.002 mM LOD, slope 4.65 ± 0.07 (au mM^-1^), R^2^ = 0.9999. The coefficient of variation (CV%) of measurements performed on the same vial was <2%; the coefficient of variation (CV%) of measurements performed on different vials was < 3%.

#### Lactate analysis by RP-HPLC with UV detection

An HPLC system (1260 Infinity Agilent Technologies) equipped with mechanical degassing system autosampler and UV/vis diode array was employed (volume injected 10 μL). Separations were carried out by a RP HPLC column Hydra RP C_18_ (Phenomenex) 250 x 4.6 mm (silica particle size 4 μm) with an isocratic elution in 100% 0.1% phosphoric acid, flowing at 0.8 mL min^-1^. The chromatographic run was complete in 15 min, after 20 injections a rinsing of the column in 100% methanol and the re-equilibration step was performed. Detection was performed at 210 nm. All the solutions were filtered using a 0.22 μm regenerate cellulose filter (Millipore, Milan, Italy). For lactate determination the dynamic linear range (DLR) was 0.03–350 mM, 0.03 mM LOD, slope 47.9 ± 0.62 (au mM^-1^), R^2^ = 0.9987. The figures of merit were comparable with those obtained previously [47]. The coefficient of variation (CV%) of measurements performed on the same vial was < 1%; the coefficient of variation (CV%) of measurements performed on different vials was < 2%.

#### Recovery

In order to evaluate the recovery 3 samples of blank CCM (5 ml each) were spiked with 0.05 mM EtOH and 5 mM lactate (final concentration) and analyzed. Recovery was 98% ± 3% and 101% ±3% for EtOH and lactate respectively.

#### Experiments to exclude EtOH contamination

After Tl exposure we found that EtOH is the main component of VOCs detected in the head space of CCM (see the Results). To rule out that EtOH was a contaminant, produced by a possible CCM degradation, due to cell-independent processes or to bacterial contamination, a number of control experiments were performed in different conditions.

First we excluded that EtOH was formed by spontaneous degradation of CCM. The flasks containing only fresh cell-free CCM were incubated at 37°C for various times, up to 25 days. Flasks were sealed, to isolate their content from the external environment, otherwise gas exchange was allowed with the incubator. The absence of EtOH in these conditions demonstrates that EtOH production requires the presence of cells.

In order to demonstrate that EtOH after Tl exposure was not produced by an eventual, undetectable, bacterial contamination of CCM, we collected 500 μL of cell-free CCM from flasks containing cell cultures exposed to Tl, in which EtOH was determined. This aliquot was added to control flasks containing 5 mL of fresh medium and incubated at 37°C up to 25 days. In these conditions the growth of any eventual bacterial clone present in the medium would produce EtOH. Since EtOH was not found in these control flasks, we can conclude that EtOH does not come from bacterial contamination. No alcohol compounds were employed to guarantee the sterility of the working area.

## Results and discussion

**Fig 1:**
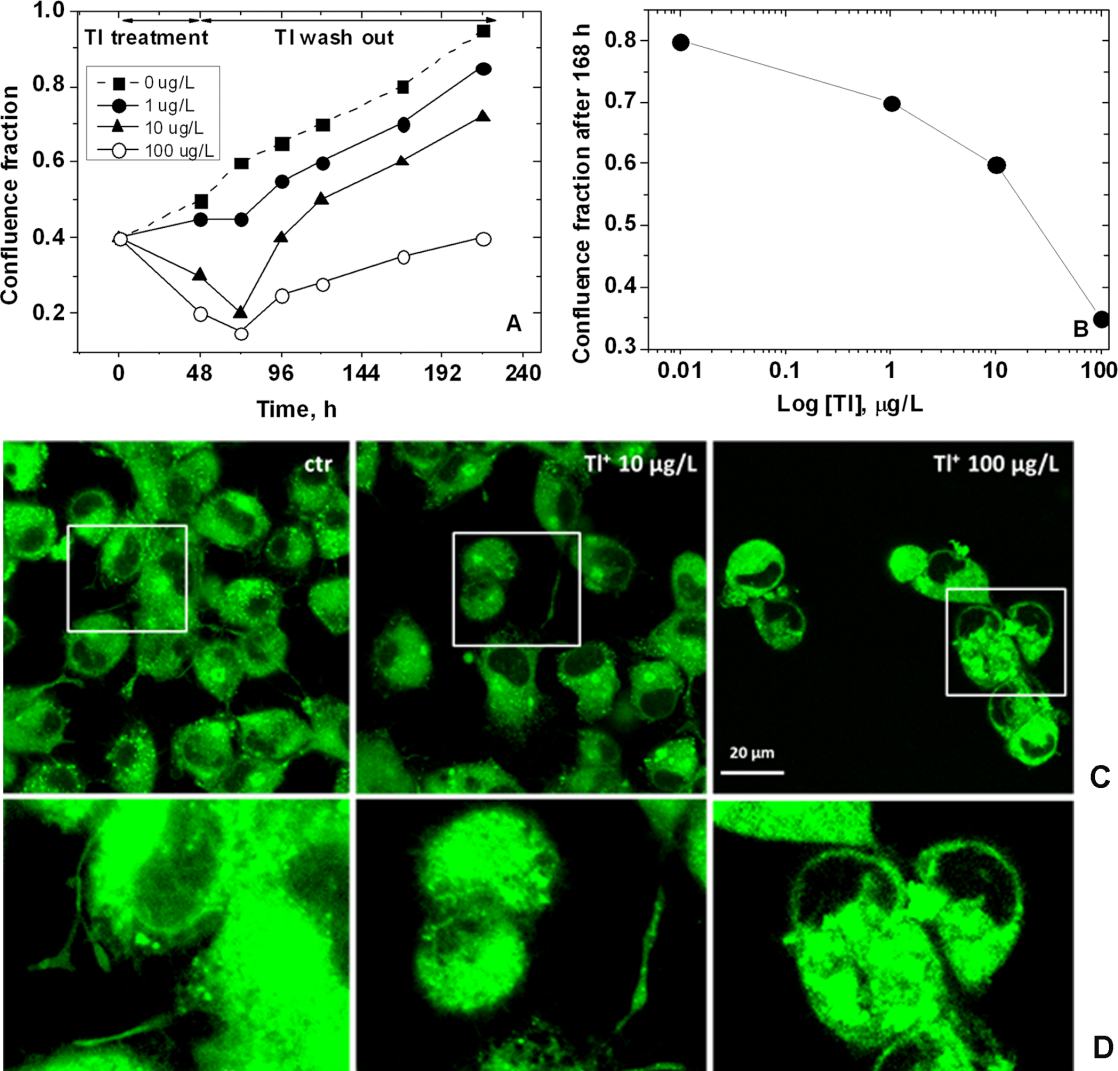
A) Tl effect on the neuronal growth, measured evaluating the confluence expressed as a fraction of cell population (SD = 2%, N = 3 independent experiments, n = 5 fields 400 x400 μm). B) Confluence fraction 5 days after the treatment *vs* Tl concentration. C) Morphology of HN9.10e neurons in controls and after 48 h exposure to 10 and 100 μg/L Tl single dose. D) Enlargement of the regions indicated in C, corresponding to control neurons with neurites (left), cells treated with 10 μg/L Tl characterized by the reduction of neurites (center) and cells treated with 100 μg/L Tl with the complete loss of neurites and the alteration of cytoplasm (rigth).

Tl exposure damaged neurons inducing alterations of morphology and confluence fraction in dose-dependent manner. Fig 1A shows the effects of Tl on the neuronal growth, evaluated as confluence fraction of the cell population. Fig 1B shows the confluence 168 h after the beginning of the experiment (48 h single dose exposure + 120 h reperfusion) *vs* Tl concentration. Fig 1C shows the morphology of HN9.10e neurons in control and after 48 h exposure to different Tl doses. Fig 1D is the enlargement of the regions indicated in Fig 1C, showing control neurons with neurites, following the definition of Flynn et al. and Dotti et al. [48, 49] (left), cells treated with 10 μg/L Tl characterized by the reduction of neurites (center) and cells treated with 100 μg/L Tl with the complete loss of neurites and the alteration of cytoplasm (rigth).

**Fig 2:**
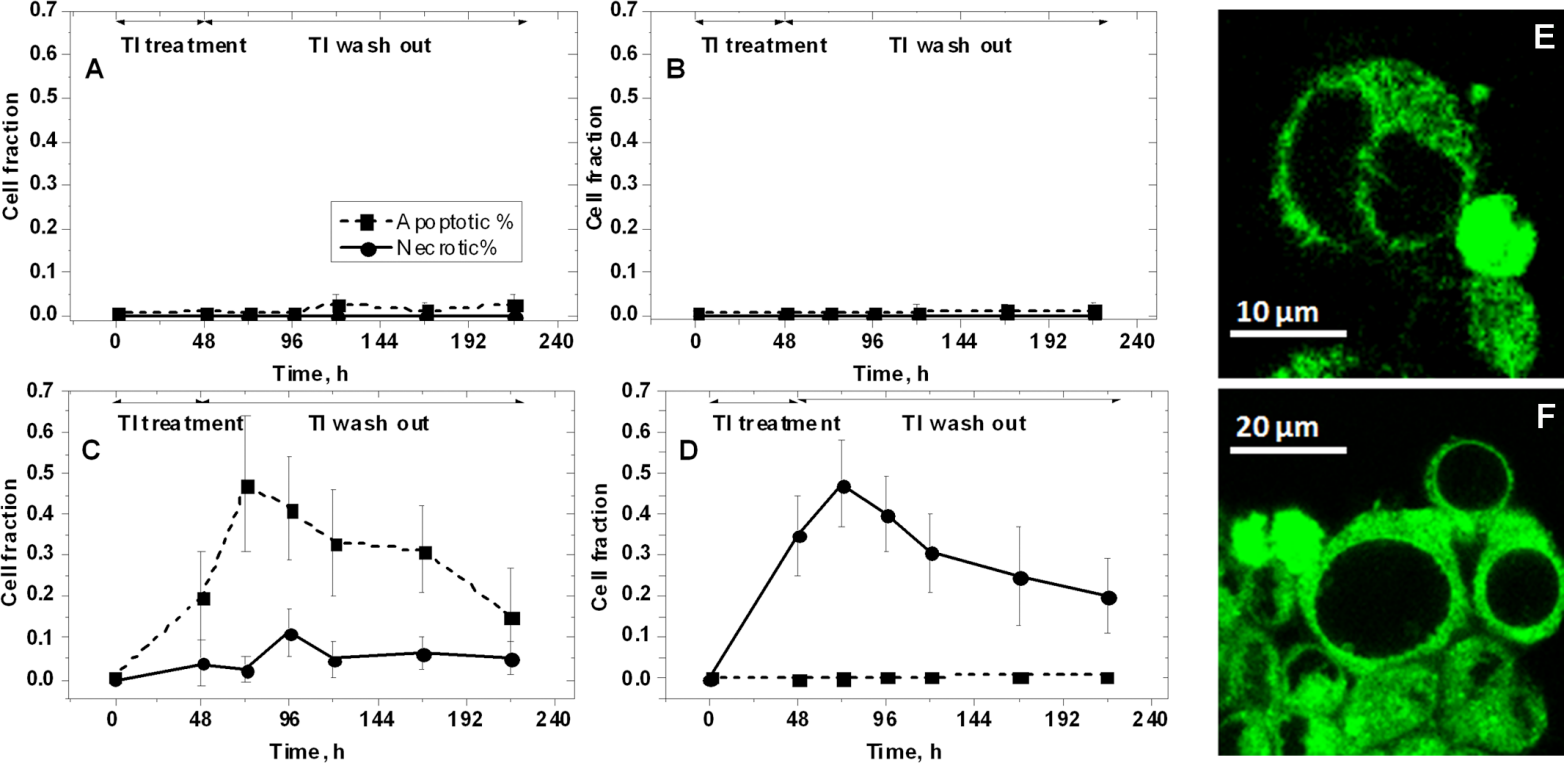
Apoptosis and necrosis incidence in cell cultures 48 h after the incubation and during the following 7 days of reperfusion: A) 0 Tl; B) 1 μg/L Tl; C) 10 μg/L Tl; D) 100 μg/L Tl. Representative images of apoptotic (E) and necrotic, swelled (F) neurons.

Fig 2 shows the apoptosis and necrosis incidence in cell cultures exposed to the different doses of Tl (A-D panels). Fig 2E and 2F show representative images of apoptotic and necrotic, swelled neurons, respectively.

The exposure to 1 μg/L Tl did not induce neuronal death (Fig 2B). However, Tl arrested the neuronal growth for at least 24 h. The gradual raise of the confluence fraction was observed in the following 2–3 days up to a full recovery (Fig 1A). The exposure to 10 μg/L Tl reduced the cell density by the induction mainly of apoptosis (up to 50%) and in lesser extent of necrosis (Fig 2C). A qualitative observation indicated that 10 μg/L Tl reduced also the number and the extension of neurites in most cells, even if no evident damage was detected in the cell body region (Fig 1D, central panel).

The neurons that survived to 100 μg/L Tl single dose appeared markedly altered and characterized by 100% loss of neurites, by alterations of cytoplasmic structure (Fig 1D, right panel) and by cellular swelling, which is the sign of osmotic unbalance (Fig 2F). In cell cultures exposed to 100 μg/L Tl we observed up to 50% cell death, exclusively due to necrosis (Fig 2D). While apoptosis is an energy-dependent cell death, necrosis does not require any energy input from the cell [50]. On this basis it is plausible to assume that the exposure to Tl single doses over 10 μg/L produces a severe metabolic dysfunction that drastically reduces the cellular energy charge, interfering with the active, energy-dependent cellular processes, including apoptosis. Also in this case, cells remained in a phase of growth arrest for at least 24 h after Tl removal from the CCM. After this phase they slowly restarted to proliferate.

Concerning the lactate and EtOH determination in CCM, the first significant observation was the presence of EtOH in CCM of cells before any treatment. The EtOH concentration found was 0.18 ± 0.013 mM, which represents about 5% of lactate concentration (3.4 ± 0.10 mM). To be certain that EtOH was not a contaminant a number of control experiments were performed (see Materials and Methods). In summary, no solvents, buffers or tubes were found to have EtOH contamination > 0.03 ± 0.003 mM and any significant possibility of contamination from swabs was also eliminated.

It is widely demonstrated that EtOH is produced by bacterial metabolism. In EtOH fermentation, one glucose molecule breaks down into two pyruvates by the action of glycolitic enzymes. Then, pyruvate is converted to EtOH and CO_2_ in two steps, catalyzed by pyruvate decarboxylase. EtOH is further converted to acetaldehyde by alcohol dehydrogenase (ADH).

In humans it has been shown that blood EtOH levels may increase in some conditions (e.g. pregnancy, diabetes, heart disease, liver disease, schistosomiasis, cryptococcal meningitis, and hypoxia) not necessarily correlated to alcohol abuse [51, 52]. Although this EtOH has been defined as “endogenous” EtOH [53], it is actually produced by bacteria present in the gut (microbiota) [54].

However, other currently undetermined metabolic processes may produce EtOH in human cells [51, 52].

The first evidence of the production of actual, endogenous EtOH in mammalian tissues was reported by McManus et al. in 1960 [55]. EtOH was detected in rat, rabbit and human tissues (liver, plasma, kidney, skeletal muscle) in amounts ranging from 23 to 145 μmole/100 mg of tissue [55].

High EtOH concentrations in the cerebrospinal fluid (CSF) and blood samples from patients under pathological conditions such as cervical myelopathy (CM) [56], amyotrophic lateral sclerosis (ALS) [57, 58], have also been reported. In CM it correlates with the severity of the disease [56].

Recently, a significant elevation of endogenous EtOH and methanol was found in the plasma of healthy women and men after intake of the specific ADH inhibitor 4-methylpyrazole (4-MP) and recently methanol-sensitive genes have been identified [59]. The inhibitor 4-MP was administered by intraperitoneal injection, which bypasses the gastrointestinal tract, in order to show that the inhibition was related to liver ADH rather than the ADH of the intestinal microflora [60].

**Fig 3:**
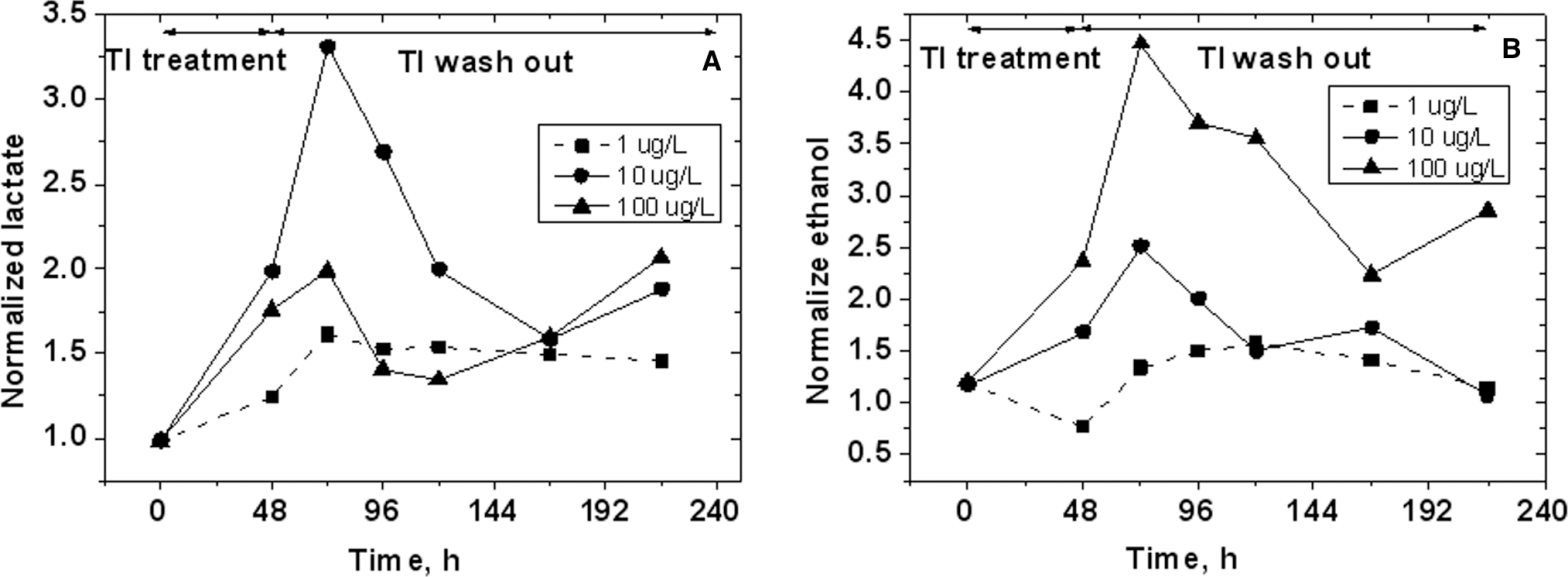
Lactate (A) and EtOH (B) concentrations normalized with respect control values found in the CCM 48 h after the incubation and during the following 7 days of reperfusion (3% SD in lactate determination; 7% SD in EtOH determination, N = 3 independent experiments). CCM was removed from the flasks for the analysis and replaced every 24/48 h after the treatment with the Tl single dose (1, 10 and 100 μg/L).

Fig 3 shows the trend of lactate and EtOH released by cells in the CCM. The data have been corrected by the confluence percentage calculated with respect to the confluence of the control culture at each single time tested and normalized with respect to the concentration values of the controls not exposed to Tl.

Lactate increased by 50% after the treatment with 1 μg/L Tl and during the wash out. The lactate in CCM of reperfused cells exposed to 10 μg/L Tl increased up to about 3.5 times the control value 24 h after the reperfusion, reaching about 4.5 mmol/L concentration, slowly decreased the following four days. Seven days after neuron reperfusion lactate started to increase again, suggesting that neurons that survived Tl exposure could change their metabolism. Further investigations on this topic are in progress.

The lactate in CCM of reperfused cells 24 h after the exposure to 100 μg/L Tl increased up to about 2 times the control value, reaching about 2.5 mM concentration, and decreased the following two days. After this lactate started to increase as observed in the 10 μg/L Tl-exposed cells.

Many evidences in the literature suggest that the intracellular accumulation of Tl in nervous tissue alters the transmembrane ion transport, the metabolism of proteins and the enzymatic reactions involved in the production of energy [23, 24, 42, 43]. However, the Tl mechanism of action is not clear [24, 61].

The metabolic switch to aerobic lactate production is known as Warburg effect [62–64]. Warburg discovered that cancer cells display a decrease of mithocondrial respiration even in the presence of oxygen and an enhanced production of lactate (named *aerobic glycolysis*) [62, 64]. It was hypothesized that mitochondria were not able to efficiently metabolize the pyruvate due the inhibition of the pyruvate dehydrogenase (PDH) complex activity and that this inhibition decreases the pyruvate transport into the mitochondrial matrix [64]. The accumulation of pyruvate in the cytoplasm induces an over activation of lactate dehydrogenase and the increase of lactate production [62–64].

The trend of EtOH in our experiments is analogous to that of lactate. However, lactate started to increase in CCM after 48 h incubation in 1 μg/L Tl, while EtOH decreased by about 25% and only 24 h after reperfusion it increased.

Furthermore, the maximum concentration of lactate (4.5 mmol/L) was found in CCM of 10 μg/L Tl-treated cells 24 h after reperfusion; the maximum concentration of EtOH (0.58 mmol/L) was found in CCM of 100 μg/L Tl-treated cells 24 h after reperfusion, while in CCM of 10 μg/L Tl-treated cells EtOH was about 0.3 mmol/L.

In summary, (i) the finding of EtOH in the CCM of neuronal cells and (ii) the increase of lactate and EtOH observed in Tl-exposed cells (Fig 3) are novel. Before the present observation, EtOH has been detected only in A549 lung cell lines [40].

The metabolic disturbance observed in our cell cultures exposed to Tl is in agreement to that observed in ALS, where the mitochondrial dysfunction is well assessed [58]. The impairment of the mitochondrial function gives the accumulation of pyruvate that, in part, is removed from the cytoplasm thanks to its conversion to lactate.

The lactate production seems to be a “safety procedure” which allows the cells to proliferate also when mitochondrial function is compromised [42] or in hypoxic environments [65]. This down-regulation of their oxidative metabolism could help these cells to escape from death [66]. However, when the damage due to the toxic agent is massive the cell likely recovers *in extremis* the energy for cell survival converting lactate in EtOH [67].

It is known that the mitochondrial pyruvate decarboxylase (EC 4.1.1.1, a thiamine pyrophosphate and magnesium-dependent enzyme) and the cytosolic ADH permits in some species of fish (including goldfish and carp) to perform EtOH fermentation (along with lactic acid fermentation) when oxygen is scarce [68]. The activity of ADH is at least 100-fold higher than that of pyruvate decarboxylase. In this way the acetaldehyde produced by pyruvate decarboxylase is not accumulated because it is fast converted into EtOH [68].

In neuronal cells ADH is not expressed and the enzymes involved in EtOH/acetaldehyde conversion are catalase and Cytochrome P450 2E1 (CYP2E1), which belongs to P450 family [59]. These enzymes are typically involved in EtOH detoxification [69]. It cannot be excluded that one or both these enzymes may work also for the production of EtOH, following the reactions:

$$Acetaldehyde+H_{2}O\overset{catalase}{\to }EtOH+H_{2}O_{2}$$ (1)

$$Acetaldehyde+H_{2}O_{2}+NADP^{+}\overset{CYP2E1}{\to }EtOH+O_{2}+NADPH$$ (2)

Enzymatic reaction (1) is compatible with the Tl-induced generation of H_2_O_2_ observed by Hanzel et al. [42] in rat adrenal pheochromocytoma cells due to the impairing of the mitochondrial function.

CYP2E1 induction in neuronal cells and the resultant oxidative stress cause apoptotic cell death in these cells, suggesting that CYP2E1 is one of the key players to target alcohol-mediated brain toxicity [70]. A possible physiological role of EtOH and its metabolites in the regulation of the growth, metabolism, differentiation and neuroendocrine function in mammals cannot be excluded [69, 71].

A disrupted mitochondrial function and “hypermetabolic and hypercatabolic state” has been frequently observed in ALS models [58, 72]. By using ^1^HNMR, a longitudinal study of CSF metabolomic signature in ALS indicated the increase of several discriminating metabolites (glucose, lactate, citric acid, ethanol) [57, 73]. The activation of glycolysis with the production of lactate during the proliferation phase has been also observed in B lymphocytes prior to their differentiation in plasma cells *in vitro* [74]. Garcia-Manteiga et al. observed, as well, EtOH in the media of differentiating primary and lymphoma B cells [74].

## Conclusions

The increase of lactate and EtOH in Tl-exposed cell cultures suggests significant alterations in energy metabolism. The experiments in HN9.10e cells herein described suggest the activation of an ancestral mechanism to survive hypoxia/mitochondrial impairment, a kind of “functional hypoxia”, in which the EtOH production is the key adaptation that allows a continued high level of glycolysis [68].

In summary, our experiments demonstrated a marked toxicity of Tl even at low doses (4.9, 49 and 490 nM) and for relatively short incubation times (48 h). Considering that Tl^+1^ rapidly distributes in the various body compartments competing with K^+^ [16], our results should give an alert on the need of review the concentration limits of Tl in the environment and in the body fluids.

## Supporting information

S1 File Graphical abstract. (DOCX)
