# Supplementary figures and images for: Thallium stimulates ethanol production in immortalized hippocampal neurons
Source: PLoS One. 2017 Nov 21;12(11):e0188351. doi: 10.1371/journal.pone.0188351 (PMC5697870; doi:10.1371/journal.pone.0188351)

**GRAPHICAL ABSTRACT**

**
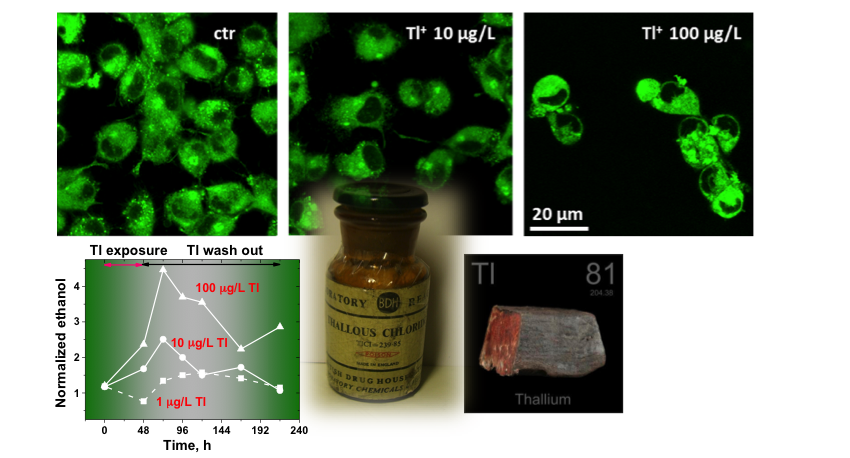
**

Supplement: S1 File — (DOCX) [file pone.0188351.s001.docx]
